# Dose–Response Relationship Between Sleep Regularity Index and Stage-Specific Alzheimer’s Disease: Cross-Sectional Evidence from Japanese Adults

**DOI:** 10.3390/geriatrics11020032

**Published:** 2026-03-18

**Authors:** Yue Cao, Jaehee Lee, Jaehoon Seol, Kenji Tsunoda, Kyohei Shibuya, Jieun Yoon, Tetsuaki Arai, Tomohiro Okura

**Affiliations:** 1 Doctoral Program in Physical Education, Health and Sport Sciences, Graduate School of Comprehensive Human Sciences, University of Tsukuba, Tsukuba 305-8574, Ibaraki, Japan; s2230494@u.tsukuba.ac.jp; 2 Department of Sport Science, College of the Arts and Sports, University of Seoul, Seoul 025504, Republic of Korea; lee.jaehee.ga@u.tsukuba.ac.jp; 3 Institute of Health and Sport Sciences, University of Tsukuba, Tsukuba 305-8574, Ibaraki, Japan; tsunoda.kenji.ga@u.tsukuba.ac.jp (K.T.); shibuya.kyohei.gm@u.tsukuba.ac.jp (K.S.); yoon.jieun.fu@u.tsukuba.ac.jp (J.Y.); 4 College of Sport Science, Sungkyunkwan University, Suwon 16419, Gyeonggi-do, Republic of Korea; 5 International Institute for Integrative Sleep Medicine (WPI-IIIS), Tsukuba Institute for Advanced Research (TIAR), University of Tsukuba, 1-1-1 Tennodai, Tsukuba 305-8575, Ibaraki, Japan; 6 R&D Center for Tailor-Made QOL, University of Tsukuba, Tsukuba 305-8550, Ibaraki, Japan; 7 Doctoral Program in Public Health, Graduate School of Comprehensive Human Sciences, University of Tsukuba, Tsukuba 305-8577, Ibaraki, Japan; 8 Department of Psychiatry, Division of Clinical Medicine, Institute of Medicine, University of Tsukuba, Tsukuba 305-8575, Ibaraki, Japan; 4632tetsu@md.tsukuba.ac.jp

**Keywords:** sleep disorder, rest–activity rhythm, optimal health, preclinical, dementia, geriatrics

## Abstract

**Background/Objectives**: Daily sleep patterns are associated with cognitive health and Alzheimer’s disease (AD). However, it remains unclear how suboptimal irregular sleep manifests in AD from the preclinical stage to dementia. This study aimed to establish the dose–response association between sleep irregularity and psychometrically defined stage-specific AD as well as executive dysfunction, among adults with subjective cognitive and sleep issues. **Methods**: Cross-sectional data were obtained from 532 Japanese adults (mean age = 63.9 years) between March 2023 and April 2024. Sleep irregularity was quantified using the Sleep Regularity Index (SRI) with 24/7 accelerometer data. A modified Poisson regression with cubic splines was performed to establish the dose–response association. **Results**: This study identified novel non-linear associations. The prevalence ratios of cognitive impairment, defined as being in the preclinical and more advanced stages of AD, significantly declined beyond a median SRI of 60. Participants within this SRI range also showed significantly lower prevalence ratios of poorer Trail Making Test B performance. All results were independent of age, sleep duration, and risk of depression. **Conclusions**: Maintaining balanced-to-regular daily sleep patterns might be optimal for AD progress from its preclinical stages, with a potential benchmark at SRI of 60, especially for those individuals at risk for cognitive decline and sleep disorders. Further research is needed to replicate this benchmark in diverse populations and to evaluate the effect of rigid sleep regularity on cognitive health.

## 1. Introduction

Despite decades of research focusing on quantitative aspects of sleep (e.g., excessively short or long sleep duration, the amount of napping [1,2,3], and qualitative aspects (e.g., poor sleep quality, the amount of slow wave sleep, and sleep fragmentation) [4,5,6], in relation to cognitive impairment and decline from the early to late stages of Alzheimer’s disease (AD), recent attention has shifted to daily sleep patterns, namely, the regularity and variability of the 24-h rest–activity rhythm [7].

Established metrics that describe rest–activity rhythm include the standard deviation of sleep timing, as well as non-parametric measures such as interdaily stability (IS), intradaily variability (IV), amplitude, and the more recently developed sleep regularity index (SRI) [8,9,10]. Although these measures differ in their specific quantification, they are likely to capture the common construct of individuals’ daily sleep patterns. More importantly, such metrics have been widely applied and are strongly linked to cardiovascular [11,12] and lifespan mortality [13,14,15].

Beyond general health outcomes, recent epidemiological evidence has shown that disrupted daily sleep patterns—measured by the aforementioned metrics—are associated with poorer cognitive performance in domains such as verbal and working memory [16,17], processing speed, and overall executive function [18,19,20]. Besides cognitive performance, these disrupted patterns have been linked to AD-related biomarkers, including β-amyloid (Aβ) deposition [21,22], the cerebrospinal fluid (CSF) p-tau/Aβ42 ratio [22,23], and brain structures [24,25,26], all of which reflect underlying AD pathology [27]. Thus, established evidence suggests that disrupted daily sleep patterns not only contribute to current cognitive deficits but also serve as a strong predictor of future AD progression [26,28,29].

However, some research gaps remain. First, although daily sleep patterns are associated with future dementia risk, when these disruptions become detectable, conversely, whether they are associated with the early stage of AD, namely, in a preclinical stage of AD (preclinical AD), or individuals experiencing subjective cognitive decline [30,31] has received little attention. While a growing proportion of individuals fall into these early and at-risk stages [32], few studies have examined this issue directly [21,22,23]. Despite the metrics applied and the slightly contradictory findings, to the best of our knowledge, SRI has not yet been examined in this context, although its potential utility in capturing daily sleep disruptions more effectively than established metrics [8].

Second, whether regular sleep patterns continuously improve cognitive health remains unclear. Although the aforementioned evidence suggests a likely linear association, one recent longitudinal study reported that individuals with highly regular sleep patterns, as measured by SRI, had an increased risk of incident dementia compared to those with more balanced sleep patterns (e.g., SRI around 60), however this trend was not statistically significant [26]. Notably, our previous work demonstrated that participants with moderate SRI levels (ranging from 55 to 65) had significantly higher serum brain-derived neurotrophic factor (BDNF) [33], which is considered protective against cognitive decline [34,35]. Also, previous studies have consistently suggested a non-linear, U-shaped association between sleep duration and AD-related pathology and cognitive outcomes, indicating the presence of an optimal range of sleep behavior in daily life [1,36]. Collectively, these findings raise the possibility of a non-linear association, whereby rigid daily sleep patterns may also be suboptimal for AD pathology. However, evidence remains limited regarding the dose–response in this context, especially the benchmark of how daily sleep patterns vary among AD stages.

Finally, while most studies have focused on either cognitive function or AD stages separately, few have combined both to capture the full spectrum of AD progression [27], specifically executive dysfunction, which tends to be a meaningful and sensitive marker emerging in the early stage of AD [37,38].

Therefore, the primary aim of this study was to establish whether sleep regularity via SRI is associated with cognitive impairment across psychometrically defined AD stages, including cognitively healthy, preclinical AD, mild cognitive impairment (MCI), and dementia, and to determine if increased sleep regularity consistently corresponds to a lower prevalence of cognitive impairment. The second aim of this study was to examine whether such associations also exist between sleep regularity and executive dysfunction.

## 2. Materials and Methods

### 2.1. Participants

All participants were research community volunteers in the Tsukuba Happiness Life Study (THLS) cohort study in Tsukuba City, Ibaraki, Japan, and provided written informed consent. This study was conducted in accordance with the principles of the Declaration of Helsinki. The THLS protocol received ethical approval from the Institutional Review Board (IRB) of Tsukuba Clinical Research & Development Organization and was registered at the University Hospital Medical Information Network on 21 June 2022 (trial ID: UMIN000051941). The registry entry was subsequently made publicly available (https://center6.umin.ac.jp/cgi-open-bin/ctr/ctr_view.cgi?recptno=R000059272, accessed on 23 January 2026). This study also received ethical approval from the Ethical Committee of the University of Tsukuba Hospital on 17 May 2023 (Approval No. R03-335). The THLS focuses on middle-aged to older adults with subjective concerns about their cognitive and sleep health, and healthy longevity, the details of which have been described previously [33]. Briefly, approximately 10,000 individuals aged from 45 to 89 years old were randomly selected by residence and invited to a mail survey in late 2022. Those who completed the mail survey and met at least one of the following inclusion criteria were enrolled in the THLS field assessment: participants reporting (1) subjective cognitive issues (based on scores of the Everyday Cognition Scale-12 ≥ 1.54 points) [39]; (2) sleep issues (based on scores of the Athens Insomnia Scale ≥ 6 points [40] and/or the Pittsburgh Sleep Quality Index (PSQI) > 5.5 points) [41]; or (3) reduced life function included one or more of the following: unintentional weight loss, reduced walking ability, or history of stroke, cardiac, or musculoskeletal disease.

During the THLS field assessment, all participants underwent a cognitive assessment battery during weekday visits and received an accelerometer (wGT3X-BT, ActiGraph, Pensacola, FL, USA) to wear 24/7 on their non-dominant wrist for the next 7 days after visits, along with a daily sleep diary to record bedtime and wake time. After 7 days, participants returned the ActiGraph and sleep diary by mail. We analyzed data from THLS participants assessed between March 2023 and April 2024, during which 599 individuals completed the field assessment.

### 2.2. Assessment of SRI, Physical Activity, and Sleep Behaviors

The SRI, physical activity, and sleep behaviors were calculated using the open-source R package GGIR (version 3.1.4) [42]. Based on data collected by the ActiGraph (sampled at 60 Hz, with 10-s epoch length). The SRI quantifies the consistency of a participant’s rest–activity rhythm by measuring the probability of being in the same sleep or wake state at two time points exactly 24 h apart across the study period (up to 7 days/168 h in our study) [10]. Higher SRI values indicated more regular or fixed sleep patterns (SRI = 100), whereas lower SRI values indicated greater irregularity or completely reflected random sleep patterns (SRI = 0). Besides the SRI, various physical activity and sleep behavior parameters were calculated using GGIR. These included sedentary behavior time, low-intensity physical activity time, moderate-to-vigorous-intensity physical activity time (MVPA), time in bed (TIB), total sleep time, wake after sleep onset (WASO), number of awakenings (NOA), sleep latency, and sleep efficiency. The threshold for physical activity was aligned with previous research [43,44]. Sleep–wake classification was determined using the Cole–Kripke algorithm implemented in GGIR [45]. Bedtime and wake time from sleep diaries were used to detect sustained inactivity bouts [46]. Sleep latency was defined as the time from TIB to the first epoch and was classified as sleep using the Cole-Kripke algorithm. WASO was defined as the duration of wakefulness after sleep onset, and NOA as the number of awakening episodes. Sleep efficiency was defined as the ratio of the total sleep time to TIB. Valid ActiGraph data for each participant were defined as wearing the device for ≥16 h a day for at least 5 days, which ensured accurate calculation of SRI and sleep behavior parameters [15].

### 2.3. Psychometric Cognitive Assessment

Participants completed the THLS cognitive assessment battery in the field assessment, as detailed in the earlier report [33], including the Trail Making Test-Japanese (TMT) [47], the Japanese version of the Logical Memory subtest of the Wechsler Memory Scale (form A) (LM) [48], and the Mini-Mental State Examination-Japanese version (MMSE) [49]. The TMT includes part A (numbers only) and part B (alternating numbers and characters from the Japanese “Hiragana” syllabary). Participants drew lines connecting 25 markers in numerical order for TMT A, and alternating numerical and alphabetical order for TMT B, without lifting their pencils. The completion times for both parts were recorded, along with two derived parameters: the B−A difference and B/A ratio [50]. The Japanese version of the Logical Memory subtest involves participants listening to a short story and immediately recalling it (LM–I), followed by a delayed recall 30 min later (LM–II). Scores were based on the number of correctly recalled items in the LM–II. The MMSE is an 11-question measure that tests five areas of cognitive function: orientation, registration, attention and calculation, recall, and language (scores ranging from 0 to 30). All assessments were administered face-to-face by trained staff using a single-blind protocol to ensure standardized data collection.

### 2.4. Classification of Cognitive Impairment by AD Stages

Participants were classified into AD stages as: cognitively healthy control (HC): healthy/possibly healthy [51]; preclinical AD: subjective cognitive decline (SCD)/objective subtle cognitive decline (OSCD)/combined of both [51,52,53]; MCI: early/non-amnestic/later [51,54]; and dementia [51,55,56]. Classification criteria was based on three components [51,52,53,54,55,56]: (1) performances of MMSE (≥24 scores or ≤23 scores) and the LM–II (with education-adjusted cut-off scores); (2) Clinical Dementia Rating (CDR) (score of 0 or ≥0.5) [57], which assigned by experienced clinicians, including dementia specialists at the University of Tsukuba Hospital based on participant’s cognitive performances and informant-reported daily functioning; and (3) self-reported memory complaints (reported/no reported). The detailed criteria for each group are summarized in Supplementary Table S1.

### 2.5. Statistical Analysis

This study primarily aimed to examine the dose–response associations between SRI and cognitive impairment in stage-specific AD. First, a modified Poisson regression was conducted with robust standard errors, incorporating cubic splines with knots placed at the 10th, 50th (median), and 90th percentiles of the SRI distribution. Continuous SRI was modeled as an independent variable, and the prevalence of cognitive impairment was used as the dependent variable (i.e., predicted outcome). The prevalence ratios (PRs) and 95% confidence intervals (95% CI) were calculated.

Second, the total sample was stratified into separate subsamples in which the predicted outcomes were defined as cognitive impairment, including preclinical AD, MCI or dementia. In the second subsample, participants with MCI or dementia were excluded, and the predicted outcome was defined as preclinical AD. In the third subsample, participants with preclinical AD were excluded, and the predicted outcomes were defined as MCI or dementia. The median SRI of each subsample was used as a reference for the modified Poisson regression model. To further confirm the non-linear associations using the regression model, continuous SRI was divided into tertiles (e.g., low, middle, and high SRI tertile groups) and included as a categorical variable. This study repeated the modified Poisson regression mentioned above in the SRI tertile analysis of the full sample and the two subsamples.

Third, to establish dose–response associations between SRI and executive dysfunction, the modified Poisson regression and tertile analysis were repeated by setting continuous SRI as the continuous independent variable, including executive dysfunction as the dependent variable. The definitions were as follows: if the TMT A, TMT B, or TMT (B−A) difference exceeded the upper quartile separately, or if the TMT (B/A) ratio was > 2.5 [58]. The same covariate adjustments were applied to the exploratory analysis. Consistent with the main analysis, an SRI tertile analysis was conducted to further establish a non-linear association.

We selected covariates based on both collinearity results and their practical explanatory significance. The final adjusted models included age, sex, body mass index, total sleep time, smoking status, alcohol consumption, years of education, Geriatric Depression Scale scores (GDS-15), self-reported economic status (poor/relatively poor/normal/relatively wealthy/wealthy), employment status (currently employed/unemployed), and partnership status (living with others/alone). Alongside covariate selection, we documented all demographic information encompassed in the THLS protocol to provide a comprehensive description of participants’ characteristics.

Statistical analyses were performed using Stata/SE (version 17.0; StataCorp LLC., College Station, TX, USA) and R statistical software (version 4.2.1). For all multiple comparisons, post hoc tests with Bonferroni correction were applied. Statistical significance was defined as a two-sided *p*-value < 0.05.

## 3. Results

Of the 599 participants who completed the THLS field assessment, 49 were excluded due to missing valid ActiGraph data, five due to invalid sleep detection, and 13 due to incomplete cognitive assessment or missing information, which prevented the collaborating clinicians from making a classification. Thus, the final analytic sample consisted of 532 participants (female, *n* = 265) with complete data on both ActiGraph-wearing and cognitive assessments (mean age = 63.9 ± 11.0 years).

Among the total 532 participants, 99 were cognitively healthy (mean age = 59.2 ± 9.5 years), 376 were in the preclinical AD stage (mean age = 64.1 ± 10.7 years), and 57 had MCI or dementia (mean age = 70.6 ± 11.3 years). Participants’ characteristics, physical activity, and sleep behaviors of the AD stage groups are shown in Table 1. Among groups, Sedentary behavior time ranged from 780.0 to 800.3 min per day, whereas low-intensity physical activity ranged from 137.3 to 156.5 min per day. Time spent in moderate-to-vigorous physical activity ranged from 60.2 to 81.0 min per day across groups. Regarding sleep parameters, time in bed ranged from 407.4 to 440.6 min, and total sleep time ranged from 329.7 to 342.3 min. Wake after sleep onset ranged from 49.3 to 73.3 min, with the number of awakenings ranging from 13.2 to 14.4 times per night. Sleep latency ranged from 24.6 to 25.4 min, and sleep efficiency ranged from 77.9% to 81.8% across cognitive groups.

Also among groups, we found that significant differences in cognitive assessment performance among the three AD stage groups, including the MMSE (*p* < 0.001), LM-II (*p* < 0.001), TMT-A (*p* < 0.001), TMT-B (*p* < 0.001), and TMT (B−A) difference (*p* = 0.003), but not in the TMT (B/A) ratio (*p* = 0.091) (Supplementary Table S2). Post hoc tests revealed significant differences in LM–II, TMT-A, and TMT-B across all three AD stage groups (all *p* < 0.05). Significant differences in the MMSE and TMT (B–A) difference were observed between the MCI or dementia group and the other two groups (all *p* < 0.05) (Supplementary Table S2).

### 3.1. SRI and Cognitive Impairment by AD Stages

**Figure 1:**
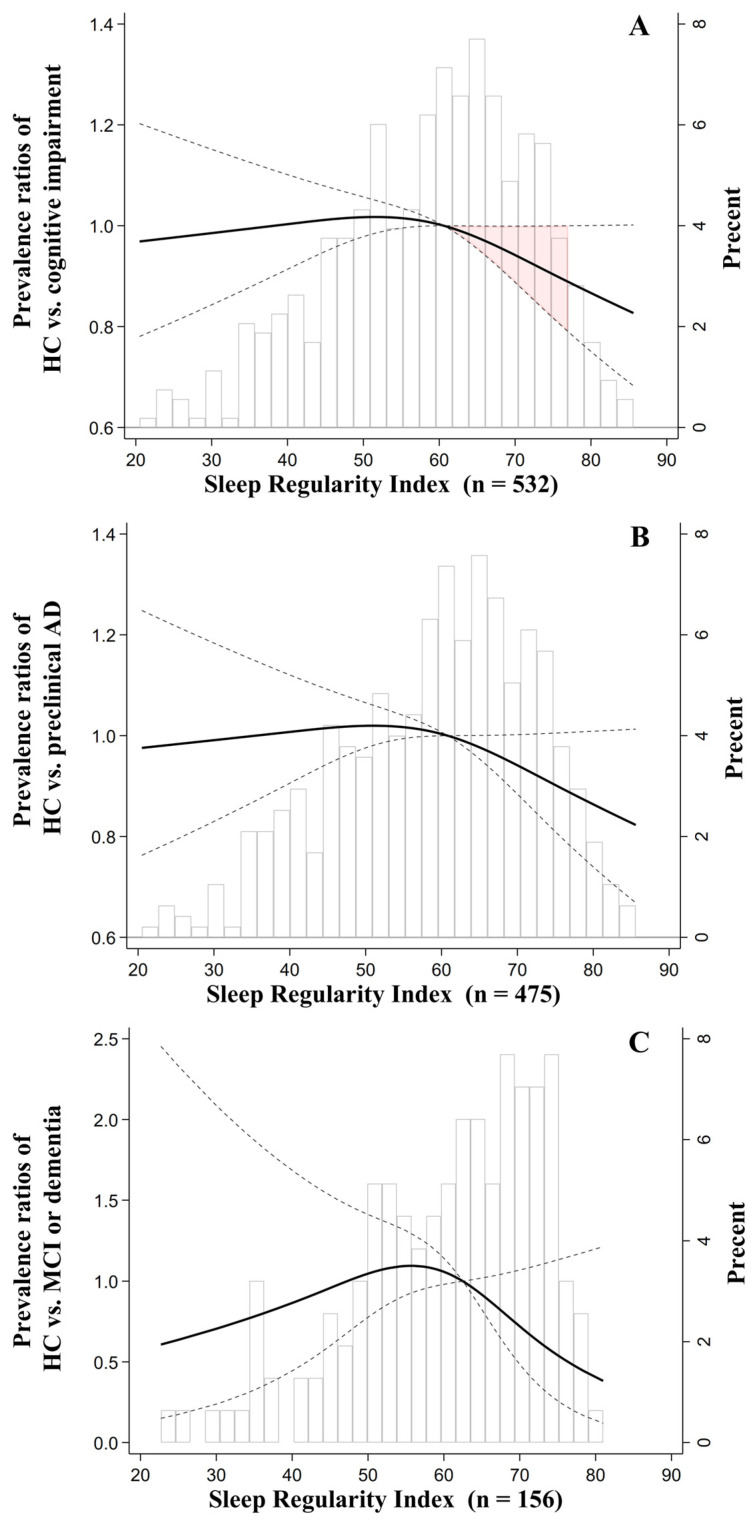
Results of the modified Poisson regression of the associations between SRI and the prevalence of cognitive impairment (**A**), preclinical AD (**B**), and MCI or dementia (**C**) after adjustment. The knots were placed at the 10th, 50th (median), and 90th percentiles, and the median SRI in each sample was set as a reference: 60.6 for (**A**), 60.8 for (**B**), and 62.5 for (**C**), respectively. The solid line represents the prevalence ratios, the dashed line represents the 95% confidence intervals, and the red-shaded area represents the regions where the CIs did not exceed 1. The bars represent histograms. For adjustment, the covariates were age, sex, body mass index, total sleep time, smoking status, alcohol consumption, years of education, Geriatric Depression Scale scores (GDS-15), self-reported economic status, employment status, and partnership status. AD, Alzheimer’s disease; SRI, Sleep Regularity Index.

In the total sample (*n* = 532), the SRI ranged from 20.5 to 85.6 with a median of 60.6. After adjusting for covariates, a non-linear association was found between the SRI and the prevalence of cognitive impairment in all AD stages (Figure 1A). The PRs remained at a plateau from low SRI to the median and then decreased significantly toward higher SRI levels; however, this decreasing trend was attenuated after an SRI of approximately 77, where CIs reached a non-significant boundary (PRs = 1) (Figure 1A). At the 10th percentile of SRI, the PRs were 1.00 (95% CI: 0.92, 1.10), and at the 90th percentile, they were 0.91 (95% CI: 0.82, 1.00). This non-linear association was confirmed in the SRI tertile analysis after adjustment, as participants in the lower (SRI range: 20.5–54.4, PRs = 1.17, 95% CI: 1.03, 1.32) and middle (SRI range: 54.5–65.6, PRs = 1.19, 95% CI: 1.05, 1.34) tertiles showed significantly higher, yet comparable, PRs compared to the upper tertile (SRI range: 65.7–85.6) (Table 2). An additional age- and sex-stratified analysis was conducted to examine further potential heterogeneity in the association between SRI and cognitive impairment (Supplementary Table S3). Among middle-aged participants (45–65 years, *n* = 291), those in the lower and middle SRI tertiles had significantly higher PRs than those in the upper tertile, and these associations remained significant after adjustment. Among older participants (>65 years, *n* = 241), a similar pattern was observed; however, the associations were attenuated after adjustment. In the sex-stratified analysis, among males (*n* = 267), participants in the lower and middle SRI tertiles had significantly higher PRs than those in the upper tertile before and after adjustment. In contrast, among females (*n* = 265), no statistically significant associations were observed between SRI tertiles and cognitive impairment.

In the second subsample (*n* = 475), where participants with MCI or dementia were excluded, the SRI ranged from 20.5 to 85.6 with a median of 60.8. The association between SRI and the prevalence of preclinical AD showed a similar shape to that of the total sample but was less pronounced and not statistically significant (Figure 1B). However, similar to the total sample, the SRI tertile analysis also revealed that lower (SRI range: 20.5–54.6) and middle (SRI range: 54.7–66.2) tertile groups had significantly higher PRs than the upper (SRI range: 66.3–85.6) tertile group, with PRs of 1.18 (95% CI: 1.04, 1.33) and 1.20 (95% CI: 1.06, 1.36) after adjustment, respectively (Table 2).

In the third subsample (*n* = 156), where participants with preclinical AD were excluded, the SRI ranged from 22.7 to 81.0 with a median of 62.5. The shape of the association between the SRI and the prevalence of MCI or dementia differed, with the highest PRs around the median SRI and lower values at both extremes. However, this trend was not significant (Figure 1C). Nevertheless, in the SRI tertile analysis, the lower (SRI range: 22.7–56.0) and middle (SRI range: 56.1–68.1) tertile groups showed more than twice the PRs compared to the upper tertile group (SRI range = 68.2, 81.0) in the unadjusted model, with PRs of 2.50 (95% CI: 1.34, 4.68) and 2.20 (95% CI: 1.16, 4.19), respectively (Table 2). After adjustment, significant differences remained only between the middle and upper tertiles (PRs = 2.64, 95% CI: 1.32, 5.28) (Table 2).

### 3.2. SRI and Executive Dysfunction

**Figure 2:**
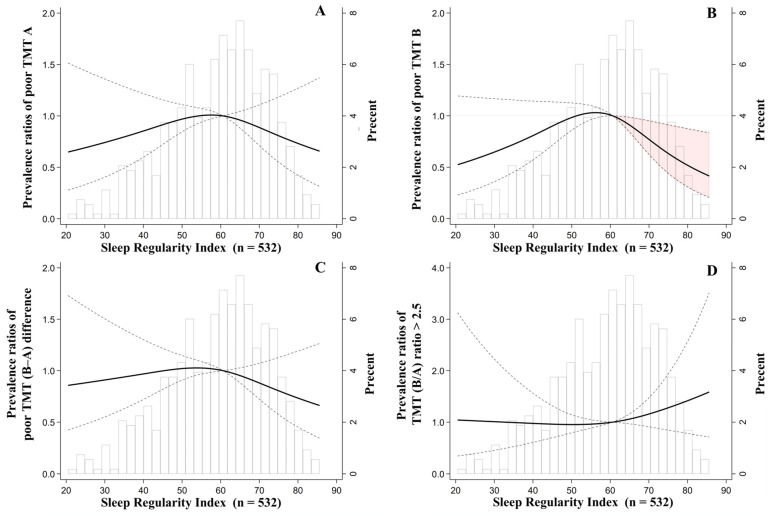
Results of the modified Poisson regression for the associations between SRI and the prevalence of executive dysfunction: poor performance on TMT A (completion time > upper quartile) (**A**), TMT B (completion time > upper quartile) (**B**), TMT (B–A) difference (>upper quartile) (**C**), and TMT (B/A) ratio > 2.5 (**D**) after adjustment. The knots were placed at the 10th, 50th (median), and 90th percentiles, and the median SRI of 60.6 was set as the reference for all the models (**A**–**D**). The solid line represents the prevalence ratios, the dashed line represents the 95% confidence intervals, and the red-shaded area represents the regions where the CIs did not exceed 1. The bars represent histograms. For adjustment, the covariates were age, sex, body mass index, total sleep time, smoking status, alcohol consumption, years of education, Geriatric Depression Scale scores (GDS-15), self-reported economic status, employment status, and partnership status. SRI, Sleep Regularity Index; TMT, Trail Making Test.

This study examined the association between SRI and executive dysfunction using the TMT performance (Figure 2 and Table 2). A higher SRI was significantly associated with a lower prevalence of poor performance on TMT, from around the median SRI (60.6) to higher values (Figure 2B). For TMT A, TMT B–A difference, and TMT B/A ratio (Figure 2A,C,D), no significant associations were observed as the CIs crossed the null. In the SRI tertile analysis, only TMT-A showed a significant difference before adjustment, with the lower tertile group having higher prevalence ratios than the higher tertile group (PRs = 1.58, 95% CI: 1.07, 2.31). However, this association was attenuated to non-significance after covariate adjustment, which was consistent with the findings for the other TMT parameters (Table 2).

## 4. Discussion

This study utilized a novel finding on how daily sleep patterns, measured using the SRI, are associated with stage-specific AD in middle-aged and older adults with subjective concerns about cognition and sleep. After adjusting for covariates, it was found that SRI had a non-linear association with cognitive impairment, including preclinical AD and advanced stages. The results showed that prevalence ratios remained relatively stable across lower SRI values and significantly decreased after reaching an SRI of 60. However, this trend plateaued at the upper end of the SRI range. This non-linear association was independent of total sleep time and was further supported by tertile analysis, in which participants in the group with an SRI of around 65 to 85 had significantly lower PRs. Interestingly, participants with an SRI above 60 also showed lower PRs of executive dysfunction based on TMT B completion time. Together, our results suggest that balanced-to-regular daily sleep patterns are associated with early cognitive changes and executive function. Therefore, based on our cross-sectional findings, an SRI of around 60 may serve as a potential benchmark for future sleep-based strategies, although a causal effect cannot be drawn from the present study.

Previous studies linking rest–activity rhythm metrics to AD-related biomarkers and AD progression yielded mixed results. For example, more pronounced sleep fragmentation, measured by IV, as well as day-to-day variability in sleep duration and sleep efficiency, are associated with Aβ burden [22,59], whereas others using similar classification approaches reported null findings for these metrics but detected greater day-to-day variability in preclinical AD compared with cognitively healthy controls [21]. Such inconsistencies likely reflect methodological heterogeneity, including differences in the choice of AD biomarkers and specific rest–activity metrics. With increasing regularity, we found that the prevalence ratios of cognitive impairment significantly decreased, beginning at an SRI of approximately 60 and continuing into a higher range (Figure 1A). These findings are consistent with our previous report that individuals with moderate SRI levels (range, 55–65) had higher serum BDNF levels [33], suggesting that neurotrophic mechanisms might partly mediate the protective effect of sleep regularity starting from moderate levels. One other recent study using public data from the UK Biobank [28] also reported a continuous protective association between SRI and incident dementia among highly regular but short sleepers (SRI > 70, sleep duration < 7 h). In our results, the statistical models were adjusted for total sleep time; however, our participants had a relatively short total sleep time of 333.5 min (i.e., mean is 333.5 min, Table 1), despite such short sleep duration being commonly observed in Japanese communities [60]. Thus, it is suggested that highly regular daily patterns might continuously contribute to cognitive health against AD progression among individuals who experience insufficient sleep time.

While comparing our findings with previous studies reporting U-shaped associations between sleep regularity and incident dementia based on longitudinal follow-up data [26], it is important to note that the study populations differed substantially in their demographic and lifestyle characteristics, including racial composition and employment status. Despite these differences, both previous evidence and our current results consistently suggest that sleep regularity may exert a protective association with AD progression beginning at moderate levels of regularity. However, longitudinal evidence has also suggested that very high sleep regularity may, in certain contexts, reflect constrained lifestyles characterized by limited social engagement, physical disability, or residence in care settings [26], which may in turn contribute to cognitive decline [61]. In contrast, our current participants were community-dwelling adults and free from long-term care services according to the THLS recruitment protocol. Nevertheless, as the maximum observed SRI in our sample was approximately 85, the potential cognitive effects associated with extremely high levels of sleep regularity could not be evaluated. These effects, therefore, warrant further investigation in larger and more diverse samples.

Moreover, among previous studies, the distribution of SRI also varies widely, even among those using similar public data [14,15,26,28,62]. While a few studies reported a similar distribution to ours (with a median SRI of 60) [14,26] (Figure 1A), others were right-skewed compared to ours [15,28,62] (the median SRI logged around 70 to 80). Despite a potential cut-off SRI of 70, defined as irregular sleep in relation to mortality risk, driven by the 0 to the lowest 20% of the total sample, has been fairly accepted [15,28,62], sleep behaviors are influenced by cultural and ethnic contexts, including not only sleep duration but also daily sleep patterns and regularity. In this context, based on the dose–response association we have found, and tertile analysis, having an SRI ≥ 60 may be positively associated with favorable cognitive health and may be considered a potential benchmark for characterizing early AD stages. However, as this study is, to the best of our knowledge, among the first to examine daily sleep patterns and cognitive health in an Asian population, future studies in populations with similar characteristics are needed to replicate and further test this benchmark.

Among our current Japanese community sample, the association between sleep regularity and cognitive impairment appeared more pronounced among middle-aged and male participants (Supplementary Table S3). Midlife has been recognized as a critical period for the accumulation of modifiable dementia risk factors [63], during which lifestyle behaviors may exert long-term influences on neurodegenerative processes. Irregular sleep during midlife may therefore represent an early behavioral factor contributing to cognitive vulnerability. Sex differences were also evident, with the association being more pronounced among males. Previous studies have reported that males tend to exhibit greater variability in sleep–wake timing and higher levels of social jetlag compared with females [64]. Greater behavioral variability in daily sleep patterns may therefore increase susceptibility to circadian disruption, potentially amplifying the cognitive consequences. Taken together, our findings suggest that maintaining regular daily sleep patterns may be particularly relevant to cognitive health in midlife, especially among males.

Beyond cognitive impairment, we also found that higher sleep regularity was continuously associated with a lower prevalence of executive dysfunction, as indicated by the TMT B completion time (Figure 2B). These results are consistent with epidemiological evidence linking disrupted rest–activity rhythms to poorer performance in domains such as processing speed and set-shifting [19,20], as well as distinguishing differences in AD stages [65,66]. However, no significant differences were found among the SRI tertile groups (Table 2), although the CIs were close to the significant boundaries (Table 2), which is plausible because the tertile approach potentially masks within-group differences in TMT B when the decline is concentrated above the median. When isolating the non-motor component of the TMT [50], by examining the B−A difference and B/A ratio, no associations with sleep regularity were observed. As these derived score indices minimize the influence of processing speed, including visual search, the null findings might suggest that sleep regularity exerts stronger effects on motor- and attention-related aspects of executive function than on set-shifting aspects.

This study had several strengths. To our knowledge, this is the first study to examine how daily sleep patterns via the SRI are related to both stage-specific AD and cognitive dysfunction, thereby extending the current understanding of disease progression. Additionally, data were derived from objective sleep measurements and clinical cognitive assessments conducted using a standardized protocol. This study also carefully adjusted for potential confounders, including age, depression, sleep duration, and current work status. Moreover, compared to previous studies that largely relied on centralized public health data, our results provide the cultural characteristics of daily sleep patterns and sleep behaviors among community-dwelling Japanese adults.

Nevertheless, our findings should be interpreted with caution due to several limitations: First, although dementia specialists confirmed all AD stage classifications, these classifications were based on assessments at a single time point, and gold-standard biomarkers such as CSF p-tau/Aβ42 ratio were not currently available. In this case, misclassification, particularly between preclinical AD and MCI, cannot be fully excluded. Second, our current data were cross-sectional, which precludes causal inference and does not rule out the possibility of reverse causation between irregular sleep and AD progression. Because cognitive impairment is defined by evidence of decline over time, a single baseline assessment may reflect inter-individual differences in cognitive reserve rather than pathological cognitive deterioration. Third, the recruitment strategy targeting individuals with subjective sleep or cognitive concerns could be considered a potential selection bias, limiting generalizability. Also, the relatively small number of participants at extreme SRI levels limits the ability to evaluate the potential effects of our current model, especially extremely regular sleep (>85), on cognitive health. Finally, potential confounding factors such as seasonal variation in sleep patterns could not be fully excluded, although participants were instructed to maintain their usual daily routines, and only valid sleep data were included. Similarly, while age was adjusted for in all models, the potential influence of aging on both cognitive health and sleep behavior cannot be entirely ruled out. Continued follow-up in the THLS cohort with a larger sample size will be important to further clarify and validate these findings.

## 5. Conclusions

It was found that sleep regularity showed a dose–response association with psychometrically defined preclinical AD, with the most pronounced decline in prevalence observed from around the median SRI onward, indicating a non-linear rather than strictly linear pattern. Our findings suggest that balanced-to-regular daily sleep patterns are associated with a lower prevalence of cognitive impairment and executive dysfunction. These associations should not be interpreted as evidence of a causal effect, and future studies need to be replicated, especially combined with longitudinal evidence to verify the proposed benchmark value of SRI 60.

## Figures and Tables

**Table 1:** Characteristics, physical activity, and sleep behaviors of participants by Alzheimer’s disease stages (*n* = 532).

Paraments	HC			Preclinical AD			MCI or Dementia		
	Total	Female	Male	Total	Female	Male	Total	Female	Male
*n*	99	56	43	376	190	186	57	19	38
Age, years	59.2 ± 9.5	57.5 ± 8.8	61.4 ± 10.1	64.1 ± 10.7	62.2 ± 10.4	66.0 ± 10.8	70.6 ± 11.3	70.2 ± 12.7	70.8 ± 10.8
BMI, kg/m^2^	22.7 ± 3.4	21.8 ± 3.2	24.0 ± 3.4	23 ± 3.2	22.3 ± 3.4	23.7 ± 2.8	23.1 ± 2.7	22.1 ± 3.3	23.6 ± 2.2
Smoking status, *n* (%)									
Currently smoking	5 (5.1)	1 (1.8)	4 (9.5)	26 (7.0)	9 (4.7)	17 (9.2)	4 (7.0)	1 (5.3)	3 (7.9)
Never quit	93 (94.9)	55 (98.2)	38 (90.5)	348 (93.1)	181 (95.3)	167 (90.8)	53 (93.0)	18 (94.7)	35 (92.1)
Alcohol consumption, *n* (%)									
Two or more times per month	54 (55.1)	26 (46.4)	28 (66.7)	201 (53.7)	79 (41.6)	122 (66.3)	25 (43.9)	6 (31.6)	19 (50.0)
Never or up to one time per month	44 (44.9)	30 (53.6)	14 (33.3)	173 (46.3)	111 (58.4)	62 (33.7)	32 (56.1)	13 (68.4)	19 (50.0)
Years of education, years	15.2 ± 3.0	14.9 ± 2.7	15.6 ± 3.4	14.8 ± 2.7	14.3 ± 2.2	15.3 ± 3.0	14.6 ± 2.9	14.0 ± 2.8	15.0 ± 2.9
Final academic degree, *n* (%)									
Middle School	3 (3.1)	1 (1.8)	2 (4.7)	11 (2.9)	5 (2.6)	6 (3.2)	3 (5.6)	1 (5.3)	2 (5.7)
High School	23 (23.5)	13 (23.6)	10 (23.3)	103 (27.5)	54 (28.4)	49 (26.5)	14 (25.9)	8 (42.1)	6 (17.1)
Vocational School	22 (22.5)	17 (30.9)	5 (11.6)	86 (22.9)	72 (37.9)	14 (7.6)	10 (18.5)	5 (26.3)	5 (14.3)
Undergraduate School	34 (34.7)	19 (34.6)	15 (34.9)	128 (34.1)	50 (26.3)	78 (42.2)	21 (38.9)	4 (21.1)	17 (48.6)
Graduate School	16 (16.3)	5 (9.1)	11 (25.6)	47 (12.5)	9 (4.7)	38 (20.5)	6 (11.1)	1 (5.3)	5 (14.3)
Employment status, *n* (%)									
Currently employed	72 (75.0)	43 (78.2)	29 (70.7)	238 (64.5)	113 (60.8)	125 (68.3)	28 (53.8)	7 (38.9)	21 (61.8)
Unemployed	24 (25.0)	12 (21.8)	12 (29.3)	131 (35.5)	73 (39.2)	58 (31.7)	24 (46.2)	11 (61.1)	13 (38.2)
Occupation, *n* (%)									
Clerical position	40 (57.1)	21 (51.2)	19 (65.5)	117 (49.6)	47 (42.3)	70 (56.0)	12 (44.4)	4 (57.1)	8 (40.0)
Business and sales	27 (38.6)	17 (41.5)	10 (34.5)	94 (39.8)	55 (49.6)	39 (31.2)	7 (25.9)	2 (28.6)	5 (25.0)
Manual laborer	3 (4.3)	3 (7.3)	0 (0)	25 (10.6)	9 (8.1)	16 (12.8)	8 (29.6)	1 (14.3)	7 (35.0)
Self-reported economic status, *n* (%)									
Poor	0 (0)	0 (0)	0 (0)	4 (1.1)	2 (1.1)	2 (1.1)	1 (1.8)	0 (0.0)	1 (2.6)
Relatively poor	7 (7.1)	4 (7.1)	3 (7.1)	36 (9.6)	18 (9.5)	18 (9.8)	7 (12.3)	4 (21.1)	3 (7.9)
Normal	56 (57.1)	34 (60.7)	22 (52.4)	240 (64.2)	124 (65.3)	116 (63.0)	31 (54.4)	7 (36.8)	24 (63.2)
Relatively wealthy	28 (28.6)	13 (23.2)	15 (35.7)	85 (22.7)	44 (23.2)	41 (22.3)	13 (22.8)	5 (26.3)	8 (21.1)
Wealthy	7 (7.1)	5 (8.9)	2 (4.8)	9 (2.4)	2 (1.1)	7 (3.8)	8 (29.6)	3 (15.8)	2 (5.3)
Partnership status, *n* (%)									
Living with others	90 (90.9)	50 (89.3)	40 (93.0)	346 (92.3)	169 (89.0)	177 (95.7)	42 (77.8)	12 (63.2)	30 (85.7)
Living alone	9 (9.1)	6 (10.7)	3 (7.0)	29 (7.7)	21 (11.1)	8 (4.3)	12 (22.2)	7 (36.8)	5 (14.3)
GDS-15, points	2.2 ± 2.0	2.4 ± 2.1	2.0 ± 1.8	3.3 ± 3.0	3.6 ± 3.0	2.9 ± 3.0	3.6 ± 3.9	3.3 ± 3.5	3.8 ± 4.1
GDS-15 points > 4.5, *n* (%)	17 (17.2)	12 (21.4)	5 (11.6)	98 (26.3)	57 (30.5)	41 (22.0)	15 (26.8)	5 (26.3)	10 (27.0)
PSQI, points	5.1 ± 2.7	5.5 ± 2.8	4.6 ± 2.6	6.0 ± 3.2	6.1 ± 3.3	5.8 ± 3.0	6.4 ± 3.7	6.5 ± 3.4	6.4 ± 3.8
PSQI points > 5.5, *n* (%)	36 (36.7)	23 (41.8)	13 (30.2)	177 (47.3)	90 (47.9)	87 (46.8)	30 (54.6)	11 (57.9)	19 (52.8)
Previous diagnoses, *n* (%)									
Hypertension	22 (22.2)	8 (14.3)	14 (32.6)	108 (28.7)	34 (17.9)	74 (39.8)	22 (38.6)	5 (26.3)	17 (44.7)
Cerebrovascular disease	1 (1.0)	1 (1.8)	0 (0)	10 (2.7)	4 (2.1)	6 (3.2)	4 (7.0)	2 (10.5)	2 (5.3)
Dyslipidemia	11 (11.1)	6 (10.7)	5 (11.6)	76 (20.2)	43 (22.6)	33 (17.7)	7 (12.3)	5 (26.3)	2 (5.3)
Diabetes	1 (1.0)	0 (0)	1 (2.3)	24 (6.4)	7 (3.7)	17 (9.1)	10 (17.5)	2 (10.5)	8 (21.1)
Respiratory disease	4 (4.0)	2 (3.6)	2 (4.7)	29 (7.7)	11 (5.8)	18 (9.7)	9 (15.8)	4 (21.1)	5 (13.2)
Sleep disorders	6 (6.1)	4 (7.1)	2 (4.7)	28 (7.5)	10 (5.3)	18 (9.7)	7 (12.3)	3 (15.8)	4 (10.5)
Sedentary behavior time, mins	780.0 ± 119.1	751.4 ± 114.2	817.2 ± 116.2	789.1 ± 109.1	768.3 ± 110.0	810.3 ± 104.4	800.3 ± 87.3	817.0 ± 122.5	791.9 ± 63.4
Low-intensity physical activity time, mins	156.5 ± 53.8	177.3 ± 51.9	129.4 ± 43.5	154.1 ± 52.6	172.9 ± 51.6	134.8 ± 46.4	137.3 ± 48.0	148.2 ± 45.0	131.8 ± 49.1
Moderate-to-vigorous-intensity physical activity time, mins	81.0 ± 36.8	81.5 ± 36.4	80.5 ± 37.8	71.8 ± 36.4	76.6 ± 38.8	66.9 ± 33.1	60.2 ± 31.2	56.1 ± 27.2	62.3 ± 33.1
Time in bed, mins	407.4 ± 70.2	400.9 ± 66.3	415.9 ± 74.9	410.9 ± 68.5	404.9 ± 67.4	417.0 ± 69.3	440.6 ± 76.8	421.8 ± 77.7	450.0 ± 75.7
Total sleep time, mins	333.5 ± 63.8	333.5 ± 60.2	333.6 ± 68.9	329.7 ± 62.5	330.6 ± 61.5	328.8 ± 63.7	342.3 ± 66.0	330.3 ± 65.9	348.3 ± 66.2
Wake after sleep onset, mins	49.3 ± 34.4	43.0 ± 29.1	57.5 ± 39.2	55.7 ± 36.6	47.5 ± 25.5	64.2 ± 43.6	73.3 ± 47.8	68.5 ± 46.6	75.6 ± 48.8
Number of awakenings, times	13.3 ± 8.0	11.3 ± 6.3	15.9 ± 9.2	13.2 ± 7.8	11.1 ± 6.4	15.4 ± 8.4	14.4 ± 7.1	11.8 ± 5.9	15.7 ± 7.4
Sleep efficiency, percent	81.8 ± 8.6	82.9 ± 8.1	80.2 ± 9.2	80.2 ± 8.5	81.4 ± 7.5	78.9 ± 9.3	77.9 ± 10.0	78.1 ± 8.6	77.8 ± 10.8
Sleep latency, mins	24.6 ± 18.5	24.4 ± 18.4	24.8 ± 18.8	25.4 ± 19.5	26.8 ± 19.8	24.0 ± 19.2	25.0 ± 15.3	23.0 ± 12.3	26.0 ± 16.8

Data are presented as mean ± standard deviation or *n* (%). Discrepancies in the totals were caused by missing data. Occupation was assessed only for participants who indicated that they were currently employed. AD, Alzheimer’s disease; HC, cognitively healthy control. MCI, mild cognitive impairment; BMI, body mass index; GDS, Geriatric Depression Scale; PSQI, Pittsburgh Sleep Quality Index.

**Table 2:** Modified Poisson regression of SRI tertiles and cognitive outcomes.

Outcomes	SRI Tertiles	Null		Adjusted	
		PRs	95% CI	PRs	95% CI
HC vs. cognitive impairment in all stages	Lower	1.21	1.08–1.37	1.17	1.03–1.32
	Middle	1.18	1.04–1.34	1.19	1.05–1.34
	Upper	(Ref)			
HC vs. preclinical AD	Lower	1.22	1.08–1.38	1.18	1.04–1.33
	Middle	1.19	1.05–1.35	1.20	1.06–1.36
	Upper	(Ref)			
HC vs. MCI or dementia	Lower	2.50	1.34–4.68	1.96	0.93–4.11
	Middle	2.20	1.16–4.19	2.64	1.32–5.28
	Upper	(Ref)			
TMT A > upper quartile	Lower	1.58	1.07–2.31	1.14	0.78–1.65
	Middle	1.45	0.98–2.15	1.40	0.96–2.03
	Upper	(Ref)			
TMT B > upper quartile	Lower	1.32	0.92–1.91	1.16	0.82–1.64
	Middle	1.16	0.80–1.70	1.30	0.91–1.84
	Upper	(Ref)			
TMT (B–A) difference > upper quartile	Lower	1.25	0.88–1.78	1.10	0.79–1.53
	Middle	0.96	0.65–1.41	1.07	0.75–1.53
	Upper	(Ref)			
TMT (B/A) ratio > 2.5	Lower	0.91	0.58–1.44	0.83	0.52–1.30
	Middle	0.72	0.44–1.18	0.74	0.46–1.18
	Upper	(Ref)			

Prevalence ratios (PRs) with 95% confidence Interval (95% CI) for the outcomes were estimated using a modified Poisson regression with robust standard errors. Adjusted models included the following covariates: age, sex, body mass index, total sleep time, smoking status, alcohol consumption, years of education, Geriatric Depression Scale scores (GDS-15), self-reported economic status, employment status, and partnership status. The upper tertile group served as the reference (set 0). AD, Alzheimer’s disease; HC, cognitively healthy control; MCI, mild cognitive impairment.

## Data Availability

The THLS dataset used in this study is available from J.Y., T.O. and the corresponding author, J.S., upon request. Inquiries regarding the study design, statistical analysis plan, and analytical code should be directed to the corresponding author, J.S.

## Supplementary Materials

The following supporting information can be downloaded at: https://www.mdpi.com/article/10.3390/geriatrics11020032/s1, Supplementary Table S1: Classification criteria for Alzheimer’s disease stages; Supplementary Table S2: Cognitive assessment results by Alzheimer’s disease stages (*n* = 532); Supplementary Table S3: Age- and sex-stratified analyses of modified Poisson regression of SRI tertiles and cognitive outcomes.

## Abbreviations

The following abbreviations are used in this manuscript:

AD	Alzheimer’s disease
BDNF	Brain-derived neurotrophic factor
CI	Confidence interval
CSF	Cerebrospinal fluid
IS	Interdaily stability
IV	Intradaily variability
MCI	Mild cognitive impairment
MVPA	Moderate-to-vigorous-intensity physical activity time
PR	Prevalence ratios
SRI	Sleep regularity index
THLS	Tsukuba Happiness Life Study
TIB	Time in bed
TMT	Trail Making Test
WASO	Wake after sleep onset
