# Supplementary material for: Dose–Response Relationship Between Sleep Regularity Index and Stage-Specific Alzheimer’s Disease: Cross-Sectional Evidence from Japanese Adults
Source: Geriatrics (Basel). 2026 Mar 18;11(2):32. doi: 10.3390/geriatrics11020032 (PMC13010776; doi:10.3390/geriatrics11020032)
Supplement: Supplementary file 1 [file geriatrics-11-00032-s001.zip › geriatrics-4141578-supplementary.pdf]

Supplementary Table S1. Classification criteria for Alzheimer's disease stages.

Stages	Criteria				*n*
	MMSE, scores	CDR, scores	LM–II, scores	Self-reported memory complaints	
HC (healthy)	≥ 24	0	≥ benchmark*	no-reported	99
HC (possibly healthy)	≥ 24	≥ 0.5	≥ benchmark	no-reported	0
Preclinical AD					
Preclinical AD (SCD)	≥ 24	0	≥ benchmark	reported	212
Preclinical AD (OSCD)	≥ 24	0	< benchmark**	no-reported	39
Preclinical AD (OSCD + SCD)	≥ 24	0	< benchmark	reported	104
MCI					
MCI (early)	≥ 24	≥ 0.5	a. 3–6 scores for 0–7 years of education.<br>b. 5–9 scores for 8–15 years of education.<br>c. 9–11 scores for ≥ 16 years of education.	reported	4
MCI (non-amnestic)	≥ 24	≥ 0.5	a. ≥ 7 scores for 0–7 years of education.<br>b. ≥ 10 scores for 8–15 years of education.<br>c. ≥ 12 scores for ≥ 16 years of education.	reported	5
MCI (later)	≥ 24	≥ 0.5	< benchmark	reported	9
Dementia***	≤ 23	–	–	–	12

There were 21 preclinical AD cases and 27 MCI cases, both diagnosed by clinicians and not further subclassified. AD, Alzheimer's disease; HC, cognitively healthy control; MCI, mild cognitive impairment; MMSE, Mini-Mental State Examination; CDR, Clinical Dementia Rating; LM–II, delayed recall of the Logical Memory subtest of the Wechsler Memory Scale; SCD, subjective cognitive decline; OSCD, objective subtle cognitive decline.

* ≥ benchmark is defined as: a. ≥ 3 scores for participants who had 0–7 years of education; b. ≥ 5 scores for 8–15 years of education; c. ≥ 9 scores for ≥ 16 years of education.

** < benchmark is defined as: a. ≤ 2 scores for participants who had 0–7 years of education; b. ≤ 4 scores for 8–15 years of education; c. ≤ 8 scores for ≥ 16 years of education.

*** Dementia was primarily classified based on MMSE ≤ 23.

Supplementary Table S2. Cognitive assessment results by Alzheimer's disease stages (n = 532).

Results	Stages			p for group
	HC (n = 99)	Preclinical AD (n = 376)	MCI or dementia (n = 57)	
MMSE, scores	29.2 ± 1.1[b]	29.0 ± 1.3[c]	27.1 ± 2.8	< 0.001
LM–II, scores	10.6 ± 3.3[ab]	8.0 ± 4.1[c]	5.5 ± 3.8	< 0.001
TMT A, seconds	29.8 ± 10.3[ab]	39.5 ± 16.1[c]	50.9 ± 22.9	< 0.001
TMT B, seconds	58.9 ± 23.7[ab]	71.6 ± 39.9[c]	98.9 ± 71.1	< 0.001
TMT (B–A) difference, seconds	29.1 ± 21.7[b]	32.1 ± 34.2[c]	49.0 ± 60.6	0.003
TMT (B/A) ratio	2.1 ± 0.8	1.9 ± 0.8	2.0 ± 1.0	0.091

Data are presented as mean ± standard deviation. AD, Alzheimer's disease; HC, cognitively healthy control; MCI, mild cognitive impairment; MMSE, Mini-Mental State Examination; LM–II, delayed recall of the Logical Memory subtest of the Wechsler Memory Scale; TMT, Trail Making Test.

Post hoc tests (Bonferroni correction applied)

[a] significant differences between the cognitively healthy and preclinical AD groups, with an adjusted $p < 0.05$.

[b] significant differences between the cognitive health group and the MCI or dementia groups with adjusted $p < 0.05$.

[c] significant differences between the preclinical AD group and the MCI or dementia groups with adjusted $p < 0.05$.

Supplementary Table S3. Age- and sex-stratified analyses of modified Poisson regression of SRI tertiles and cognitive outcomes.

Outcomes	SRI tertiles	Null		Adjusted	
		PRs	95% CI	PRs	95% CI
Middle-aged (age = 45-65 years, *n* = 291)					
HC vs. cognitive impairment in all stages	Lower	1.31	1.09–1.57	1.30	1.08–1.56
	Middle	1.00	1.04–1.47	1.04	1.05–1.54
	Upper	(Ref)			
Older (age > 65 years, *n* = 241)					
HC vs. cognitive impairment in all stages	Lower	1.12	1.00–1.26	1.06	0.93–1.21
	Middle	1.17	1.05–1.30	1.13	1.00–1.26
	Upper	(Ref)			
Female (*n* = 265)					
HC vs. cognitive impairment in all stages	Lower	1.11	0.95–1.29	1.11	0.94–1.30
	Middle	1.09	0.94–1.27	1.09	0.94–1.27
	Upper	(Ref)			
Male (*n* = 267)					
HC vs. cognitive impairment in all stages	Lower	1.31	1.10–1.56	1.27	1.06–1.51
	Middle	1.28	1.07–1.53	1.27	1.06–1.52
	Upper	(Ref)			

Prevalence ratios (PRs) with 95% confidence Interval (95% CI) for the outcomes were estimated using a modified Poisson regression with robust standard errors. In the age-stratified analysis, adjusted models included the following covariates: age, sex, body mass index, total sleep time, smoking status, alcohol consumption, years of education, Geriatric Depression Scale scores (GDS-15), self-reported economic status, employment status, and partnership status. The upper tertile group served as the reference (set 0). In the sex-stratified analysis, the same covariates were included except for sex. HC, cognitively healthy control.
